# Impact of a natural rubber-based scratcher as an environmental enrichment on the scratching behavior, cortisol level, and semen quality of stable male goats

**DOI:** 10.14202/vetworld.2024.2443-2450

**Published:** 2024-11-05

**Authors:** Sakdichod Kimsakulvech, Prarom Sriphavatsarakom, Sunsaneeya Thaikoed, Waraluk Oupala, Chainarong Punkong, Phirom Prompiram, Somchai Saingkaew, Orachun Hayakijkosol, Tuempong Wongtawan

**Affiliations:** 1 Department of Pre-Clinic and Applied Animal Science, Faculty of Veterinary Science, Mahidol University, Salaya, Nakhon Pathom, Thailand; 2 Animal Behavior and Animal-human Interaction Research Group, Akkhararatchakumari Veterinary College, Walailak University, Tha Sala, Nakhon Si Thammarat, Thailand; 3 Pasupalun Livestock and Wildlife Hospital, Faculty of Veterinary Science, Mahidol University, Sai Yok, Kanchanaburi, Thailand; 4 Department of Conservation Research and Animal Health, Khao Kheow Open Zoo. Bangpra, The Zoological Park Organization, Chonburi, Thailand; 5 The Monitoring and Surveillance Center for Zoonotic Diseases in Wildlife and Exotic Animals, Faculty of Veterinary Science, Mahidol University, Salaya, Puthamonthon, Nakhon Pathom, Thailand; 6 Division of Tropical Health and Medicine, College of Public Health, Medical, and Veterinary Sciences, James Cook University, Queensland, Australia; 7 Center for One Health, Walailak University, Tha Sala, Nakhon Si Thammarat, Thailand

**Keywords:** cortisol, goat, para rubber, scratcher, semen quality

## Abstract

**Background and Aim:**

Goats are valuable livestock because they can generate meat and milk for human consumption. Goat husbandry is becoming more intensive due to the growing demand for goat products, which may impact animal welfare and natural behavior. This study aimed to investigate the impact of natural rubber (para rubber)-based scratchers as an environmental enrichment on scratching behavior, cortisol levels, and semen quality in stable bucks (male goats/goats).

**Materials and Methods:**

Nine male goats were used in this study. Scratching behavior and cortisol levels were used as welfare indicators, whereas semen quality was evaluated as an indicator of reproductive potential. These indicators were analyzed before and after scratcher installation.

**Results:**

After installing the scratchers, the goats showed a significant increase in scratching behavior and a notable decrease in cortisol levels (p < 0.001). Notably, the goats exhibited a marked preference for scratching against the scratcher (p < 0.001) compared to the stable. They significantly preferred using their heads for scratching (p < 0.001) instead of other body parts. In addition, goats preferred to scratch on the softest rubber scratchers at specific installation locations (p < 0.001). Although there was a slight improvement in semen quality, there was no statistically significant difference (p > 0.05).

**Conclusion:**

A natural rubber-based scratcher can increase scratching behavior and reduce cortisol levels, indicating its potential to improve the welfare of farm goats. Selecting an appropriate hardness and preferred location is essential to ensure that the scratcher effectively encourages animals to use it.

## Introduction

Goats are an increasingly important livestock industry, producing milk and meat, as evidenced by the consistent increase in goat production globally [1, 2]. Intensive commercial systems [2, 3] and reproductive technology have continuously developed to increase goat production to meet high demand. Artificial insemination (AI) technology is widely used for rapid genetic improvement and production enhancement. The technique involves various steps, including semen collection, processing, and evaluation, with an emphasis on bucks (male goats/goats) [4]. Keeping male goats together at a semen collection station can increase stress and lead to aggressive behavior and territorial disputes because they are naturally territorial animals and often establish dominance hierarchies within a flock, leading to fights and injuries [5, 6].

To achieve high productivity, intensive farming is commonly implemented in enclosed barns with large groups of animals. Such environments can disrupt animals’ natural behavior and cause distress [3]. It has been illustrated that stress has profound negative effects on the productivity and reproduction of livestock, mainly through the release of stress-related hormones, such as corticosteroids [7, 8]. Stress also significantly impacts the welfare of animals, which is currently one of the most concerning aspects of the livestock industry, partly because of consumer and political demands [9, 10]. Welfare-focused practices can reduce stress levels in farm animals, such as cattle, chickens, and pigs, resulting in improved performance and productivity [11–14].

Various enrichment items have been used in livestock to improve animal welfare. For example, mechanical or motorized rotor brushes have been installed in ruminant farms, such as cattle, goats, and sheep. This apparatus mimics grooming behavior and can thus reduce animal frustration and stress [15]. This device positively affects behavior by reducing non-activity periods and increasing eating time, which then results in higher weight gain [16]. In addition, the apparatus can be used to monitor an individual’s health, as unwell animals (such as those with metritis and lameness) usually decrease their activities and, therefore, visit the apparatus less [17, 18]. Using such an apparatus could also indirectly positively affect milk production and udder health [19].

Unlike large ruminants, only a few commercial enrichments exist for goats in commercial housing [20]. In weaned kids, structural enrichment, such as ladders and bridges, significantly increases concentrated feed consumption, bipedal stance, and resting behavior and decreases abnormal oral activities; however, it does not affect the growth rate [21]. Indoor goats may exhibit stereotypic behavior that can be diminished by installing enrichment structures such as tree trunks, suspended tires, and plastic bottles [22].

Scratching is one of the most common behaviors in wild and captive goats. This activity is likely to comfort the animal, particularly to relieve itching [3]. Moreover, the rubbing behavior of animals may serve different functions, including hygiene (i.e., to remove external parasites), sensual pleasure, self-grooming, and scent deposition [23–25]. Farm goat scratch objects include stables and scratchers (in-house and commercial). Goat scratchers are usually made of hard materials such as wood, plastic, or metal, which can cause injury to the goat’s skin [26]. Therefore, replacing these traditional hard materials with softer materials, such as rubber, could potentially minimize the risk of such damage.

This study aimed to investigate the impact of a natural (para rubber)-based scratcher on cortisol levels, scratching behavior, and semen quality in stabled male goats. Cortisol levels and scratching behaviors were examined as indicators of stress and welfare, whereas semen quality was evaluated as a marker of reproductive potential. We also investigated whether goats prefer the hardness of the para rubber used in scratchers.

## Materials and Methods

### Ethical approval

This study was approved by the Institutional Animal Care and Use Committees of Mahidol University (protocol approval number: MUVS-KA-2019-09-02).

### Study period and location

The study was conducted from December 2019 to July 2020. The animal experiment was performed at the Faculty of Veterinary Science, Mahidol University, Kanchanaburi campus, Thailand. The hormone analysis was done at Khao Kheow Open Zoo, The Zoological Park Organization, Chonburi, Thailand. The behavior analysis with recorded video was conducted at Akkhararatchakumari Veterinary College, Walailak University, Nakhon Si Thammarat, Thailand.

### Animals

**Figure-1:**
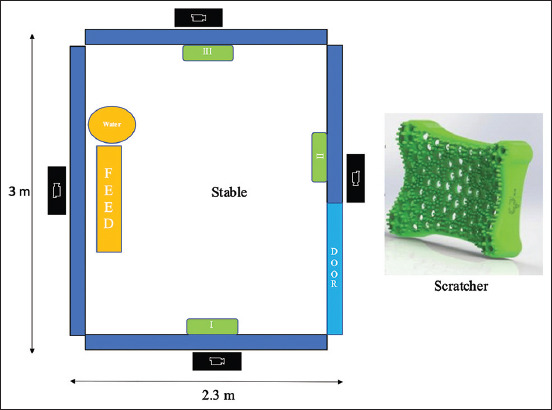
An illustration of a testing area for behavior. A test stable was equipped with CCTVs (the camera symbol) installed on each side of the stable and three scratchers (green) with different hardness values (A, B, and C) at three different locations (I, II, and III) inside the stable.

Nine mixed-breed (native × Boer) male goats (*Capra aegagrus hircus*), aged between 3 and 4 years and weighing 30 kg–45 kg, from the University’s AI station, were used in this experiment. They were fed dry Napier grass and commercially concentrated food twice a day with *ad libitum* water. They were dewormed every 3 months and received vaccines for foot and mouse disease, anthrax, and black leg disease. All goats ate normally and were physically healthy before and during the experiment. Each goat was housed in a stable-sized 2.3 × 3 × 1.5 m (width × length × height) (Figure-1) at the animal unit. Semen was routinely collected once in a month. The animals were kept in cages without rubber scratchers 1 month before the installment of scratchers.

### Para rubber

This natural rubber-based scratcher was made from para rubber (*Hevea brasiliensis*) by S.C.16 Company Ltd. (Bangkok, Thailand), in collaboration with the Rubber Technology Research Center. The size of the scratcher was 40 × 55 × 12.5 cm (width × length × depth) (Figure-1).

Three different hardness levels (Shore Hardness Score) of the rubber were tested: Shore 50 (the softest rubber, rubber A), Shore 55 (the medium-soft rubber, rubber B), and Shore 60 (the hardest rubber, rubber C). Shore hardness scores of 50 and 60 were analogous to the hardness of the erasers and tires, respectively. The scratchers were installed at three different locations (I, II, and III) inside the test stable (Figure-1) at a height of approximately 60 cm, measured from the ground to the lower border of the scratcher. The locations of these rubber scratchers were counter-balanced, switching every 4 weeks (28 days) to avoid the location effect (Table-1). Each goat was tested for 3 months. All scratchers were thoroughly cleaned with a detergent to remove any remaining scents.

**Table-1:** Sequence and location of the rubber installed at the stable.

Location	Location		

	I	II	III
Rubber sequence 1:(Week 1–4)	A	B	C
Rubber sequence 2:(Week 5–8)	C	A	B
Rubber sequence 3:(Week 9–12)	B	C	A

A=The softest rubber, B=Medium soft rubber, C=The hardest rubber

### Scratching behavior analysis

The behaviors of the tested goats were recorded daily by 4 CCTVs (Kowa, BestCCTV Ltd., Thailand). An illustration of the test cage is presented in Figure-1. Behavior analysis was performed using a continuous sampling technique with Solomon coder software (https://solomon.andraspeter.com/). The ethogram of the observed behavior is shown in Table-2. The behavior was observed for 24 h from 00:01 to 23:59 a.m. Scratching behavior was analyzed for its frequency and duration, and the sum of scratching behavior over the last day (day 28) for each subject was used in the statistical analysis. We selected the last day for observation and analysis to allow the animals to become accustomed to the scratcher. Only one researcher performed the behavioral analysis to avoid bias in the analysis

**Table-2:** Ethogram used for the goat behavior analysis.

Keyword	Description
Scratching	The goat uses any body part to rub with an object
Head scratching	The goat uses the head, face, nose, or horn to rub an object
Neck scratching	The goat uses neck rubbing with an object
Body scratching	The goat uses its thorax or abdomen for rubbing with an object
Rump scratching	The goat uses its rear end of the body to rub against an object
Leg scratching	The goat rubbing its legs against an object
Rubber A	The goat uses any part of its body to rub against rubber A
Rubber B	The goat uses any part of its body to rub against rubber B
Rubber C	The goat uses any part of its body to rub against the rubber C
Stable	The goat uses any part of its body to rub against the stable
Other objects	The goat uses any part of its body to rub against other items apart from the scratcher and the stable

### Cortisol levels

The cortisol level was used as an indicator of stress and a welfare marker [27, 28]. Blood was collected twice: once before the installation (day 0) of the para rubber scratcher and once after the use of the scratchers (day 84). A 5-mL blood sample was obtained from the external jugular vein of each goat at 09:00 am. Blood was collected by a veterinarian and one animal caretaker gently restraining the goat. The blood collection procedure for each goat lasted less than one minute to reduce stress. The serum was separated by centrifugation and transferred to a laboratory for cortisol level measurement using enzyme immunoassays, as previously described by Brown *et al*. [29] and Mesa-Cruz *et al*. [30]. Each 96-well plate (Nunc-Immuno™ Maxisorp™ Surface; Fisher Scientific, PA, USA) was coated with a rabbit cortisol R4866 polyclonal antibody (against cortisol-3-carboxymethyloxime linked to bovine serum albumin) (Coralie Munro, University of California, Davis, USA) and incubated at 4°C overnight. Different concentrations of the standard cortisol (hydrocortisone) (Sigma-Aldrich, MO, USA) and test samples were added to the wells. Horseradish peroxidase (HRP)-labeled cortisol (1:20,000) (Coralie Munro, University of California) was added and incubated at room temperature (15°C) for 1 h. The plates were washed 5 times using a wash solution before adding 2,2’-azino-di-(3-ethylbenzthiazoline sulfonic acid (ABTS) as substrate solution (Sigma-Aldrich) and incubated at 15°C with shaking for 15 min. The plate reading was executed at 405-nm wavelength. The cortisol level was measured in triplicate within the same assay.

### Assessment of semen quality

Semen quality was assessed before and after the installation of the rubber scratchers. Semen was collected using an artificial vagina in the presence of non-estrus female goats. The libido score was determined by measuring the copulation time, which was defined as the duration from the moment a male goat visually identified a female until ejaculation [31]. A libido score of 5 indicates that the male can copulate and ejaculate within 1 min of seeing a female. In contrast, a score of 0 indicates a complete lack of sexual desire, with the male not engaging in copulation with the female even after 10 min.

Assessment of semen quality encompassed the measurement of various parameters, including semen volume, sperm mass movement, motility, viability, and concentration. Semen volume was quantified using an autopipette. Mass spermatozoa movement was evaluated using a light microscope (Zeiss KF2-ICS, Germany) at a magnification of 400×, and the assessment score ranged from 0 (indicating no motility) to 5 (representing excellent motility) [32].

The percentage of spermatozoa motility was determined as the proportion of sperm cells exhibiting forward movement when observed under a light microscope at 400× magnification. Sperm concentration was assessed using a Neubauer counting chamber (Boeco, Hamburg, Germany) under a light microscope (Nikon, Tokyo, Japan), with the sperm cells fixed in formalin before counting. Sperm viability was analyzed using the hypo-osmotic swelling test, as outlined in the methodology described by Fonseca *et al*. [33]. The semen was diluted in a hypo-osmotic swelling solution (125 mOsm) at 1:400, and the viable sperms (bent-tail sperm) were counted out of 200 total sperms under a light microscope (Nikon) at 400× magnification [34].

### Statistical analysis

Scratching behavior was quantified as an average and presented as the mean ± standard deviation (SD), encompassing both frequency (time) and duration (seconds). Scratching behavior was examined in a comparative analysis between the rubber scratcher and the stable and between the day (06:01–18:00) and night (18:01–06:00) using a pair student’s t-test.

In addition, the comparison extended to various levels of rubber hardness, distinct locations, and diverse body areas where animals scratched, including the head, neck, body, and rump. These comparisons were statistically analyzed using repeated measures analysis of variance with Tukey’s *post hoc* test.

In this study, we compared the cortisol level, semen quality, and scratching behavior before and after using a rubber scratcher. Cortisol levels and semen quality were assessed by comparing the values (mean ± SD) using a paired t-test.

Statistical analyses were performed using an open software, the Jamovi version 2.2.5 [35]. p < 0.05 was considered statistically significant.

## Results

### Scratching behavior

Before the installation of the rubber scratchers, the goats often rubbed the cage with their body parts. The average frequency of rubbing against the cage was 108.33 ± 26.02 times/day, and the average duration of rubbing against the cage was 1,913.66 ± 334.15 s/day. After the installation of the rubber scratchers, the goats significantly increased the frequency of scratching behavior to 228.89 ± 34.28 times/day (t(16) = 8.39, p < 0.001) and the duration of scratching to 3,827.31 ± 668.30 s/day (t(16) = 7.68, p < 0.001).

The average frequency of rubbing against the para rubber scratcher was 191.50 ± 36.48 times/day, which was significantly higher (t(16) = 11.86, p < 0.001) than that of rubbing against the stable, 37.39 ± 13.68 times/day. The duration of rubbing against the para rubber (3,094.98 ± 676.83 s/day) was significantly longer (t(16) = 9.27, P < 0.001) than that against the stable (732.33 ± 354.50 s/day).

The goats used para rubber scratchers more frequently during the day. The average frequency of rubbing occasions at night was 57.67 ± 39.31 times/day, which was significantly lower (t(16) = 4.23, p < 0.001) than during the daytime (220.78 ± 108.77 times/day). Similarly, the average duration of rubbing the scratcher at night was 1,066.42 ± 735.01 s/day, which was significantly reduced (t(16) = 5.60, p < 0.001) compared with the daytime (2,911.07 ± 658.63 s/day).

Considering the frequency (F(3) = 79.4, p < 0.001) and duration (F(3) = 85.7, p < 0.01) of using body parts to rub against rubber scratchers, the goats demonstrated a preference for using specific organs to scratch the rubber surface. Head scratching against the rubber was statistically more frequent and longer in duration compared with other body parts, followed by the body, neck, and rump (Table-3); the *post hoc* analysis is presented in Table-4.

**Table-3:** The body parts of goats used for scratching objects.

Variables	Head	Neck	Body	Rump
Frequency (times/day)	178.22 ± 48.41^a^	4.00 ± 1.94^c^	59.56 ± 23.96^b^	0.67 ± 2.00^d^
Duration (s/day)	3,316.44 ± 934.64^a^	59.78 ± 60.63^c^	508.67 ± 376.6^b^	0.89 ± 2.67^d^

^a-c^Represents statistical differences between columns; F (3) = 79.4, p *<* 0.01

**Table-4:** *Post hoc* comparisons among the organs used for scratching.

Comparison	Mean difference	SE	df	t	p_tukey_

RM factor 1					
Head					
Neck	174.22	16.310	8.00	10.68	< 0.001
Body	118.67	19.597	8.00	6.06	0.001
Rump	177.56	16.417	8.00	10.82	< 0.001
Neck					
Body	−55.56	7.821	8.00	−7.10	< 0.001
Rump	3.33	0.928	8.00	3.59	0.029
Body					
Rump	58.89	7.560	8.00	7.79	< 0.001

SE=Standard error, df=Degrees of freedom

### Preferences of rubber hardness and location

**Figure-2:**
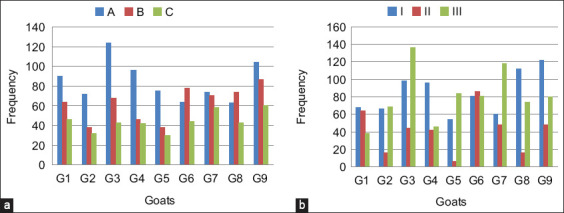
The preference of the scratcher (a) hardness and (b) location for each individual goat. Hardness; A= the softest, B = medium, C= the hardest. Location; I, II, and III are the different locations.

The goats demonstrated a statistically significant preference for the hardness of the rubber (F(2) = 18.8, p < 0.001) and the location of the installation (F(2) = 7.18, p < 0.001), as presented in Table-5. The *post hoc* analysis of the hardness preference is presented in Table-6. The results showed that the goats exhibited significantly more frequent scratching and spent significantly more time on rubber A (softest), followed by rubber B (medium-soft), and rubber C (hardest). The scratcher hardness trends of individual goats are also shown in Figure-2. Most goats (77.78%, n = 7/9) tended to prefer the softest rubber; others preferred medium-soft rubber. No goat preferred the hardest rubber.

**Table-5:** The preference of goats for rubber scratchers was based on hardness and position.

Variables	Hardness			Location		
	
	A	B	C	I	II	III
Frequency (times/day)	84.67 ± 20.44^c^	62.61 ± 17.83^b^	44.22 ± 10.01^a^	84.11 ± 24.06^b^	41.11 ± 25.34^a^	80.67 ± 30.96^b^
Duration (s/day)	1516.47 ± 549.03^c^	973.62 ± 368.07^b^	604.89 ± 217.14^a^	1,117.89 ± 238.88^b^	605.91 ± 393.43^a^	1,541.40 ± 743.63^b^

The number represents the average frequency and duration (mean ± standard deviation). ^a-c^Represent significant difference, (F (2) = 18.8, p *<* 0.001) in hardness and (F (2) = 7.18, p *<* 0.001) in location

**Table-6:** *Post hoc* comparisons between the preference for hardness of scratcher reading to frequency.

Factor	Mean difference	SE	df	t	p_tukey_
A					
B	22.0	8.41	8.00	2.62	0.071
C	40.4	6.78	8.00	5.97	<0.001
B					
C	18.4	3.76	8.00	4.90	0.003

SE=Standard error

Table-7 presents the *post hoc* analysis results for location preference. The results revealed that the goats scratched significantly more frequently at locations I and III than at location II and spent significantly more time at locations I and III than at location II. In addition, the trend in individual goat preference for scratcher locations is illustrated in Figure-2. Most goats tended to prefer either location I (n = 4/9) or location III (n = 4/9) over location II (n = 1/9).

**Table-7:** *Post hoc* comparisons among the preference for scratcher location according to duration.

Factor	Mean difference	SE	df	t	p_tukey_
A					
B	543	248	8.00	2.19	0.133
C	912	174	8.00	5.24	0.002
B					
C	369	136	8.00	2.70	0.063

SE=Standard error

Notably, no skin injury was observed during the experiment, and the scratcher did not show prominent damage after three months of use.

### Cortisol level

The mean serum cortisol level of male goats before installing the para rubber scratchers was 14.98 ± 6.31 ng/mL, whereas the mean serum cortisol level after using the rubber scratcher for 3 months was 2.58 ± 2.60 ng/mL. This reduction was observed to be statistically significant (t(8) = 6.10, p < 0.001).

### Semen quality

Table-8 details the tested parameters for semen quality. The presence of scratchers slightly increased sperm motility, mass movement, concentration, and viability, albeit with a decrease in semen volume. However, no statistically significant differences were observed (t(8) = 0.24, p > 0.05) for any parameters.

**Table-8:** Semen quality before and after using para rubber sheet.

	Semen volume (mL)	Mass move	Motility (%)	Concentration (×10^6^)	Viability (%)
Before	0.64 ± 0.25	4.13 ± 1.13	71.88 ± 15.10	7,022.50 ± 3,011.43	54.38 ± 25.27
After	0.48 ± 0.23	4.25 ± 0.71	79.38 ± 14.25	7,795.00 ± 1,937.03	61.25 ± 25.34

## Discussion

The findings of this study highlight the beneficial impact of the para rubber-based scratcher on male goats, suggesting its potential as an environmental enrichment tool to promote natural scratching behavior, alleviate stress, and positively influence reproductive physiological parameters.

Although the total length of the scratcher was 165 cm shorter than the length of the metal structure around the cage (895 cm) in the absence of para-rubber, the goats in this study exhibited a clear preference for rubbing their bodies against the para-rubber scratchers over the stable structure. In addition, the increased scratching behavior observed after installing the rubber scratchers confirmed the preference for rubber scratchers over cages. Notably, scratching behavior can serve as a means of seeking comfort when experiencing stress or pain [36, 37]. Stress can be caused by keeping goats in cages all the time, as our study revealed high cortisol levels before installing the scratcher and decreased cortisol levels after having the scratcher.

In this study, the cortisol level was used as an indicator of stress and a welfare marker, as shown in many species in which high cortisol levels are associated with stress [27, 28]. The changes in serum cortisol levels in our study were similar to the fluctuations observed in serum cortisol levels during seasonal or transportation stress [38–40]. Specifically, the lower serum cortisol levels after being introduced to the scratchers were similar to the reduction in the cortisol levels detected in the goats during autumn compared with that during winter, which was presumably caused by distress from the cold weather [38, 39]. An increased cortisol level can also be observed during transportation [40]. This study suggests that stress in stable male goats, as measured by plasma cortisol levels, can be effectively alleviated by rubbing a rubber-based scratcher.

Considering the preference for rubber hardness, this study revealed that the goats preferred to scratch on the softest rubber. It is worth noting that the hardness preference may vary among animal species. For instance, cattle tend to favor the hardest scratchers [41], whereas horses prefer softer scratchers (Wongtawan, unpublished data). Para rubber is an attractive material option due to its natural properties, flexibility, and strength, making it a safer choice for animals than plastic or wood. Prioritizing animal safety and well-being when selecting scratcher materials. These results imply significant benefits for physical and mental health of animals, as the rubber scratcher reduces stress and skin damage.

In addition to the preference for rubber hardness, the choice of installation location for a scratcher is also crucial. In this study, we observed that goats preferred specific scratch locations. This location-dependent scratching behavior has also been noted in various species, including cattle, horses, chimpanzees, and cats [41–43]. The preference for the scratching location in these goats may be attributed to a left-right bias, as the scratcher was positioned to the left and right of the feed tray. The current study revealed that most goats preferred either the left or right side of the feed tray (n = 8/9). In contrast, only one goat preferred the rubber positioned in the opposite direction from the feed tray. The phenomenon of left and right bias or preference has been observed in various species, including dogs, cats, horses, cattle, and goats [44–46].

Regarding scratching behavior, this study revealed that goats use their heads significantly more often than other body parts when interacting with scratchers. This behavior might suggest a purpose related to scent deposition, as the cephalic (head) region is known to be one of the primary glandular areas in the ungulates [47]. It is plausible that the rubber scratcher enhances communication among goats by providing an additional means of olfactory signaling. This aspect of the head rubbing against the scratcher deserves further examination to better understand its functional significance in communication.

Regarding semen quality, no significant changes were observed before and after the installation of the para rubber scratchers. This is consistent with a previous study by Hoyer [48] on bulls, which found that access to a swinging cow brush did not significantly change semen quality. It is worth noting that both studies employed some bulls for their investigations, which reduced the statistical power, making it harder to identify significant effects or relationships. However, exposure to stress and elevated cortisol levels can negatively affect semen quality in male goats [49, 50]. Therefore, installing a scratcher could be beneficial in the long term because it has been shown to reduce cortisol levels in male goats, as demonstrated in this study.

One primary limitation of this study was the small sample size of male goats due to most goats in Thailand are female and the availability of male goats capable of producing semen for AI is restricted, posing challenges in terms of acquiring a substantial number of male goats for the experiment.

## Conclusion

This study demonstrated that male goats favor para rubber-based scratchers because they scratch the rubber more frequently than stable animals and exhibit increased scratching behavior after installation. Goats also exhibited a preference for the softest rubber in specific locations. Selecting the optimal hardness and preferred location for the scratcher is crucial for encouraging animals to use it. The installation of rubber scratchers significantly reduced the cortisol level, indicating the potential to reduce stress and improve welfare.
